# Comparison of Intracytoplasmic Sperm Injection Outcomes between Oligozoospermic, Obstructive Azoospermic and Non-Obstructive Azoospermic Patients 

**Published:** 2012-06-19

**Authors:** Ensieh Shahrokh Tehraninejad Tehraninejad, Elham Pourmatroud, Mohammad Ali Sadighi Gilani, Mahdi Rakebi, Zahra Azimi Neko, Arezoo Arabipoor

**Affiliations:** 1 Department of Endocrinology and Female Infertility, Reproductive Biomedicine Research Center, Royan Institute for Reproductive Biomedicine, ACECR, Tehran, Iran; 2 Faculty of Medicine, Tehran University of Medical Science, Tehran, Iran; 3 Department of Andrology, Reproductive Biomedicine Research Center, Royan Institute for Reproductive Biomedicine, ACECR, Tehran, Iran

**Keywords:** ICSI, Obstructive Azoospermia, Oligozoospermia

## Abstract

**Background:**

To determine the differences in sperm quality and results of intracytoplasmic sperm injection (ICSI) cycles between three groups of male factor infertile couples: oligozoospermic, obstructive azoospermic and non-obstructive azoospermic.

**Materials and Methods:**

In this prospective cohort study, 628 male factor infertile couples who underwent ICSI cycles from April 2004 to March 2006 were enrolled. Three hundred fourteen oligozoospermic patients (group I), 180 obstructive azoospermic patients (group II) and 134 non-obstructive azoospermic patients (group III) were included. Fertilization, cleavage, implantation and clinical pregnancy, early abortion rates were assessed. Chisquare and analysis of variances with Post Hoc (Tukey test) were used for data analysis.

**Results:**

Fertilization rates were significantly different in the three groups (group I: 66.6%; group II: 51.8%; group III: 47.7%; p=0.004). There were differences in the implantation rates (I: 19.5%; II: 17.6%; III: 6.4%; p=0.001). The cleavage rates were found to be 55.1% (group I), 47.5% (group II), 45.5%(group III), respectively. The clinical pregnancy rate was the lowest in the third group (I: 37.6%; II: 28.9%; III: 13.4%; p=0.001). There was no significant difference in early abortion rates between the three groups: (I: 10.7%; II: 9.8%; III: 8%; p=0.776).

**Conclusion:**

It can be concluded that patients with oligozoospermia may benefit the most from ICSI treatment. ICSI cycles which use spermatozoa from non-obstructive azoospermic patients have a lower chance for successful outcome. The results of this study suggest, in cases of failure to achieve pregnancy after 1 or 2 cycles in non-obstructive azoospermic patients, embryo donation would be a better alternative.

## Introduction

Today, we know that male factor is the only cause of infertility in 20% of infertile couples, but it may be a contributing factor in as many as 30-40% of cases (1). Over the past two decades, presentation of surgical methods for sperm retrieval and intracytoplasmic injection has been achievements in the treatment of azoospermia (2). One absolute indication for intracytoplasmic sperm injections(ICSI) is severe male factor infertility demonstrated by total motile sperm count of 0.5×106/ml or less than 3% normal morphology according to Tygerberg strict criteria. Azoospermia (absence of spermatozoa in ejaculated semen) is found in about 5% of all infertile couples (3). Most azoospermic patients suffer from primary testicular failure (non-obstructive azoospermia) and show a germ cell aplasia (sertoli cell-only), a maturation arrest, or tubular sclerosis and atrophy at their testicular histopathology (4). When testicular biopsy shows a normal spermatogenesis or a mild hypospermatogenesis, an obstruction of the excretory ducts is present. Obstructive azoospermia (OA) is diagnosed when no spermatozoa are found in the pellet after centrifugation, or when only immotile nonviable spermatozoa are observed. Surgical sperm recovery is indicated in order to avoid performing ICSI with DNA-damaged spermatozoa (5).

In obstructive azoospermic patients, sperm is obtained by microsurgical epididymal sperm aspiration (MESA) or percutaneous epididymal sperm aspiration (PESA). In non-obstructive azoospermia, sperm is obtained from the testis. Open microsurgical testicular sperm extraction (TESE) yields the greatest number of sperm (5).

Various factors may affect the outcome of ICSI in azoospermic patients. These include parameters related to the male partner, such as age, serum follicle stimulating hormone (FSH) level and testicular histology that may reflect upon the quality of the surgically retrieved sperm cells. The injected sperm quality and viability may be related to the cause of azoospermia (obstructive or non-obstructive), the location of sperm origin (epididymis or testis), that shows the developmental stage of the sperm cell (6).

A meta analysis found no difference in the rate of fertilization, implantation or ongoing pregnancy between epididymal and testicular sperm for patients with the same diagnosis (7) but according to deficient meiosis and higher rate of aneuploidy in embryos, a significant lower fertilization and implantation rate were observed in couples with non obstructive azoospermia (NOA) (8).

Yet, the difference between quality of ejaculated sperm and surgically retrieved sperm (epididymal or testicular) is debatable. There are few published articles comparing the outcomes of ICSI between epididymal or testicular retrieved sperm and ejaculated sperm. Some reports are available which show no difference (2, 9), but some studies revealed better results obtained from severe oligoastheno teratospermic patients than azoospermic patients (10, 11). The objective of this study is to compare the ICSI outcomes between three groups of male factor Iranian infertile couples (oligozoospermic patients, non-obstructive and obstructive azoospermic patients).

## Materials and Methods

This prospective cohort study consisted of 628 ICSI cycles which were performed from April 2004 to March 2006 in Department of Endocrinology and Female Infertility of Royan Institute, Iran. Approval for the study was obtained from the Institutional Review Board. Signed consent forms were obtained from all patients before enrolling in the study.

In this study, all female were healthy and between the ages of 20-35. Only male factor infertility cycles were studied. The patients had a comprehensive history taken followed by physical examination, including inguinoscrotal examination. Testicular volume was determined by a Prader orchidometer and the status of the epididymis and presence or absence of vas deferens was reported. A hormonal assay [luteinizing hormone (LH), FSH and thyroid function tests] and two semen analyses within a two month interval were performed. All men with total sperm counts less than 10 million/ml were placed into group I (oligozoospermia, 314 patients); whereas, azoospermic patients were sub-grouped into two groups, according to their FSH level, testicular volume and PESA sampling. PESA was performed using a 23-gauge butterfly needle attached to a 20 ml plastic syringe which served as an aspiration device. If the results were normal (FSH>7.6 IU/ml), the patients were placed into group II (obstructive azoospermia, 180 patients) and if the results were abnormal, they were placed into group III (non-obstructive azoospermia 134 patients).

Ovulation induction was performed using routine long protocol of GnRH agonist suppression with buserelin (Superfact: Aventis Pharma Deutshlan, Frankfurt, Germany), which was subcutaneously applied in the amount of 0.5mg, started on day 21 of the previous cycle followed by stimulation with daily doses of 150-300 IU of human menopausal gonadotrophin (Menopur or HP-HMG, Ferring Co, Germany). Human chorionic gonadotrophin 10000 U (hCG; Choriomon; IBSA) was used when two or more ovarian follicles presented with a mean diameter of 18mm. Ultrasound guided transvaginal follicular aspiration was performed under general anaesthesia 34-36 hours after the hCG administration. The total number of oocytes retrieved in a cycle was 10 or more. Mechanical oocyte denudation was applied to remove all the surrounding cumulus and corona cells; afterword, nuclear maturation assessment was performed using an inverted microscope to allow injection only in metaphase II oocytes.

In group I, sperm was obtained by ejaculation after 48-72 hours of sexual abstinence, and the sample was kept at 37 ˚C for 30 minutes or until complete liquefaction. After washing and centrifugation, ICSI was performed on sperm retrieved from the epididymis or testis of groups II and III under intravenous anaesthesia (per the protocol) in association with spermatic cord blockage with 2% xylocaine in the operating room. For PESA, a 13.5 gauge needle connected to a 1 ml syringe was used and in group III, a microsurgical guided open biopsy was performed. After sperm preparation, microinjection was performed. The injected oocytes were transferred to closed culture system and incubated for 16-18 hours at 37 ˚C and 5.5% CO_2_ until fertilization was detected. Embryo transfer was performed on day 3 of ovum retrieval. The number of embryos transferred was 2 to 3 per cycle.

Fertilization, cleavage, implantation, clinical pregnancy and early abortion rates were the main outcomes evaluated. Fertilization was considered normal when 16-18 hours after injection two pronuclei (2 PN) were observed. Embryo transfer was performed 72 hours after ICSI. Luteal support included daily intramuscular injections of progesterone (100 mg). Serum βhCG was checked two weeks later. If it was positive, luteal support continued until 12 weeks. Two weeks after testing positive, vaginal ultrasound was done to assess pregnancy. Clinical pregnancy, including sonographic demonstration of gestational sac was determined. Outcomes were compared between the three groups.

Statistical analysis was performed using Statistical Package for Social Science version 16.0 (SPSS Inc., Chicago, IL, USA). Categorical data were expressed as number and percentage, and numerical data as means ± SD. When a quantitative analysis of the data was performed, groups were compared by analysis of variance using post hoc analysis. Chi-square test was applied in the qualitative analysis of the data. The chi-square test was used to compare pregnancy and early abortion rates. Differences were considered significant at p<0.05.

## Results

A total of 628 patients were included in the study. Table 1 shows the demographic and clinical characteristics of the participating couples. Patients’ ages were similar among the three groups. ANOVA test showed that duration of infertility had significant difference between oligozoospermic versus NOA patients (p=0.01).

Statistical analysis revealed significant differences in the number of oocytes retrieved between oligozoospermic versus OA patients (p≤0.001). Also, Post Hoc (Tukey) test showed meaningful differences between oligozoospermic versus OA patients as well as NOA versus OA patients in the number of embryos transferred per cycle (p<0.05) (Table 2).

**Table 1:** Patients’ characteristics in three study groups


	Oligozoospermic	Obstructive azoospermic	Non-obstructive azoospermic	P value
	(n=314)	(n=180)	(n=134)	

**Male age (Years)**	34.4 ± 5.5	34.64 ± 6.7	33.75 ± 5	0.363
**Female age (Years)**	29.03 ± 4.5	28.29 ± 4.9	28.49 ± 4.5	0.197
**Infertility duration (Years)**	6.64 ± 5	6.4 ± 4.5	5.30 ± 4.6	0.01*


* Significant difference between oligozoospermic vs. NOA patients.

ANOVA test showed meaningful differences in the mean number of grades B and C embryos between the oligozoosperm group and the two other groups (p≤0.001), but there were not significant differences in the mean number of grades A and D embryos between the three groups (Table 2).

The chi-square test demonstrated that there were significant differences in fertilization rates between the three groups, which these differences were between oligozoospermic versus NOA patients and NOA versus OA patients (p≤0.004) (Table 3).

Cleavage, implantation, and pregnancy rates also showed indicative differences among the three groups (Table 3). Chi-square test demonstrated no significant differences between the three groups in early abortion rates.

We used Backward Binary logistic regression for adjusting the effect of potential confounders only infertility duration (OR=1, 95% confidence interval (CI)=1.003-1.077, p=0.048) and sperm origin (Oligo, OA, NOA) (OR= 1.2, 95% CI=0.992-1.511, p=0.05) had effect on pregnancy rate.

**Table 2:** Comparison of ICSI cycles outcomes in three study groups


		Oligozoospermic	Obstructive azoospermic	Non-obstructive azoospermic	P value¤
		(n=314)	(n=180)	(n=134)	

**No. of oocytes retrieved/cycle**		6.4 ± 4.3	8 ± 4.4	7.4 ± 4.8	0.001*
**No. of embryos transferred/cycle**		2.7 ± 1.3	3 ± 1.2	2.7 ± 1.4	0.03*
**Quality of transferred embryos**	A	1.7 ± 1.2	1.5 ± 1.1	1.7 ± 1.1	0.14 ^a^
	**B**	0.77 ± 1.04	1.13 ± 1.18	0.76 ± 0.81	0.000 ^b^*
	**C**	0.18 ± 0.44	0.38 ± 0.87	0.33 ± 0.72	0.03 ^c^*
	**D**	0.03 ± 0.15	-	0.03 ± 0.17	0.08 ^d^


¤ ANOVA test with Post Hoc (Tukey test). * Sgnificant difference. a Oligozoosperm vs. OA (p =0.14), Oligozoosperm vs. NOA (p =0.99), OA vs. NOA patients (p=0.3). b Oligozoosperm vs. OA (p =0.01*), Oligozoosperm vs. NOA (p =0.99), OA vs. NOA patients (p=0.005*). c Oligozoosperm vs. OA(p =0.004*), Oligozoosperm vs. NOA (p =0.07), OA vs. NOA patients (p=0.7). d Oligozoosperm vs. OA (p =0.08), Oligozoosperm vs. NOA (p =0.67), OA vs. NOA patients (p=0.03).

**Table 3:** Comparison of ICSI cycles results in three study groups


	Oligozoospermic	Obstructive azoospermic	Non-obstructive azoospermic	P value¤
	(n=314)	(n=180)	(n=134)	

**Fertilization rate**	66.6%	61.4%	51.8%	0.004*
**Cleavage rate**	55.1%	47.7%	45.5%	0.01*
**Implantation rate**	19.5%	17.6%	6.4%	0.001*
**Clinical pregnancy rate**	37.6%	28.9%	13.4%	0.001*
**Early abortion rate**	10.7%	9.7%	8%	0.776


¤ Chi-square test. * Significant difference.

## Discussion

The results of this study showed that there were significant differences in the fertilization, cleavage, implantation and pregnancy rates following ICSI using surgically retrieved sperm (PESA for obstructive azoospermia; TESE for non-obstructive) and ejaculated sperm, but early abortion rates were not different between the three groups. The highest rate of ICSI outcomes was observed in the wives of oligozoospermic husbands.

Other researchers have reported the same outcomes as our study (10-12). On the other hand, Aboulghar et al. (13) and other authors did not find any significant difference between the treatment results following ICSI with ejaculated sperm and surgically retrieved sperm (2, 14, 15). However a meta-analysis of surgical sperm retrieved in azoospermic patients concluded that sperm origin does not affect cycle outcome (7), but our results suggested that sperm origin can affect cycle outcome.

In the present study, highest fertilization and cleavage rates were noted in the oligozoospermic group, but the number of occytes retrieved was the lowest in this group. Also, the numbers of embryos transferred per cycle weren’t homogenous between the three groups; the obstructive azoospermic group with the highest number of embryos transferred per cycle had the lowest implantation and pregnancy rates. Since all women in our study were under 35 years of age and the highest quality embryos were transferred one possible explanation for these findings could be perhaps that sperm quality has greater importance than both the number of oocytes retrieved and embryos transferred per cycle in predicting ICSI outcomes.

The results of this study showed that there was no significant difference between the three groups in early abortion rate. Other researchers have reported that the use of testicular sperm leads to a higher spontaneous miscarriage rate (16, 17). On the other hand, Naru et al. (2) and other researchers did not find any significant difference in the early abortion rate following ICSI with ejaculated sperm and epididymal sperm (2, 14, 18). We concluded that other factors in addition to sperm origin also affected early abortion rate.

In our study, in cases of obstructive azoospermia, PESA was initially attempted in all patients. Only those in whom no sperm cells were detected in the epididymal sample were further subjected to TESE. Therefore, when we compared outcomes between OA (group II) and NOA (group III), higher ICSI outcomes have been found in the OA group. In contrast to earlier studies (11, 19) which demonstrated lower fertilization rates in NOA patients with similar quality of embryos, implantation rates and clinical pregnancies; in our study the differences between the two azoospermic groups was not only in the fertilization rates, but also in cleavage, implantation and clinical pregnancies rates. Our results were in agreement with the Dozortsev et al. study and support their hypothesis that a motile sperm cell randomly taken from the epididymis has a lower developmental potential than a random sperm cell taken from the testicle (20). Also, according to several studies, despite normal FSH level and testis size; chromosomal abnormality and y chromosome micro deletion are higher in NOA men, so sperm retrieval and success are lower in infertile men (21).

One point of our work was the comparison between three groups of infertile men of which only a few papers have been published (2, 12). In an article by Ghanem et al. (12) 313 ICSI cycles were studied between three groups [triple defects (OAT), OA and NOA] which demonstrated significantly lower fertilization rates with ejaculated sperm (OAT) or testicular sperm (NOA); however, spermatozoa from OA patients fertilized as well as normal or mild-moderate deficient ejaculated sperm with the same embryo quality, cleavage and pregnancy rates. In our study, there was a better result uptake with oligozoospermic patients. As mentioned, DNA-damaged sperm is more in samples which are taken from testicular biopsies rather than ejaculated sperm in men with mild to moderate deficient spermatogenesis. This may explain less motile sperm found in testis biopsy with lower implantation and clinical pregnancy rates in these groups. We suggest, in OA patients, chromosomal abnormalities in sperm retrieved are less than NOA patients. Of course, a karyotype study or y chromosome fluorescent in situ hybridization (FISH) study would be necessary for a better explanation; if other semen parameters like motility and morphology could be included, the results could be more helpful in order to advise this group of NOA men to use their sperm for a maximum of one or two cycles.

## Conclusion

The present study confirms that patients with oligozoospermia may benefit the most from ICSI. Therefore, this treatment method should be considered as a high-priority technique for the treatment of oligozoospermic patients. In our culture, the use of donor spermatozoa is forbidden; therefore, ICSI for the treatment of severe male-factor infertility is especially important. Intracytoplasmic sperm injection in combination with PESA and TESE is an effective method and can successfully be performed to treat men with azoospermia, but the outcomes with these procedures are not comparable to ICSI using ejaculated sperm. ICSI cycles, which use spermatozoa from non-obstructive azoospermic patients, have a lower chance for success. If there was failure to achieve pregnancy after 1-2 cycles then sperm or embryo donation would be a better solution.
